# Effects of amniotic membrane suspension in the rat alkali burn model

**Published:** 2011-02-05

**Authors:** Jin A Choi, Jun-Sub Choi, Choun-Ki Joo

**Affiliations:** 1 Department of Ophthalmology and Visual Science, St. Vincent hospital, College of Medicine, the Catholic university of Korea, Seoul, Korea; 2 Department of Ophthalmology and Visual Science, Seoul St. Mary’s hospital, College of Medicine, the Catholic university of Korea, Seoul, Korea

## Abstract

**Purpose:**

The main objective of this study is to determine the anti-inflammatory effect of topical amniotic membrane (AM) suspension on corneal alkali burn compared to topical serum eyedrops.

**Methods:**

Thirty eyes from 30 Sprague-Dawley male rats were used. After alkali injuries using 1 N NaOH, the control group (n=10) received topical PBS four times a day for 2 days. The first study group received topical 30% AM suspension, and the second study group (n=10) received topical 30% rat serum. Using slit lamp biomicroscopy, injured corneas were evaluated and scored in terms of re-epithelialization, corneal opacity, and neovascularization (NV). Tissue sections were analyzed histologically for cellular infiltration, and immunohistochemical staining was conducted using rat anti-mouse F4/80 antibody for the detection of macrophages.

**Results:**

In the inflammatory wound healing model, the epithelial healing ratios of the control group, the AM suspension group, and the serum eyedrop group were 1.8±15.1%, 34.1±17.7%, and 41.5±16.1%, respectively (p<0.0001). The opacity scores for the control group, the AM suspension group, and the serum eyedrop group 48 h after the insult were 4.8±0.5, 3.4±0.5, and 3.0±0.8, respectively, showing a significant difference (p<0.0001). Moreover, the NV scores for the control group, AM suspension group, and serum eyedrop group 48 h after the insult were 5.8±0.9, 4.0±1.3, and 4.3±0.9 (p=0.006). Upon immunohistochemical evaluation using F4/80, significantly fewer F4/80+ cells were recruited in the AM suspension and serum eyedrop groups than the control group (p=0.027).

**Conclusions:**

The suspension form of the amniotic membrane promoted epithelial healing and reduced corneal opacity and NV in alkali burn. It also suppressed F4/80 expression in the corneal stroma, indicating that the AM suspension maintains its beneficial biochemical effect on inflammatory corneal wound healing in vivo.

## Introduction

The ocular surface and associated adnexal structures are currently recognized to form an integrated functional unit that is critically important for optical clarity, vision, protection, and ocular health [1]. These substructures interact with one another and participate actively in the defense of the ocular surface through immune cells and via cytokine release. Failure of this ocular surface can occur in a broad range of clinical conditions, with differing pathogeneses; a variety of endogenous and exogenous precipitating factors, the most common of which are chemical trauma, infection, inflammation, and hereditary conditions; or secondary ocular surface failure [2]. Some researchers have recently proposed that the mechanisms underlying neurogenic inflammation may involve the local release of neuromediators in diseases such as allergic conjunctivitis and dry eye; this phenomenon may also play a pivotal role in both the initiation of the immune response and the regulation of the chronicity of the inflammatory response [3]. Thus, in the treatment of ocular surface disease, anti-inflammatory effects, as well as rapid epithelialization, would be critical.

The use of amniotic membrane (AM) as a graft for ocular surface reconstruction was initially reported by Kim and Tseng in 1995 [4], and the popularity of this surgical procedure has increased in recent years. It has been determined that the transplantation of AM as a temporary or permanent graft promotes epithelial wound healing and exerts potent anti-inflammatory and antiscarring effects on the ocular surface [1,5]. However, despite the valuable effects of AM, the clinical application of AM transplantation is currently limited to severe cases of ocular surface disease, as these tend to require an invasive surgical procedure that may result in a variety of suture-related complications [6]. Additionally, the biochemical effect of AM may not be maintained continuously following AM transplantation. In this regard, if the suspension type of AM is provided and retains its own beneficial effect, it may prove valuable in the treatment of ocular surface disease, as AM has been shown to promote corneal epithelialization and to suppress inflammation on the ocular surface.

The principal objective of this study was to determine the anti-inflammatory effects of a topical AM suspension on a corneal alkali burn relative to topical serum eyedrops.

## Methods

### Amniotic membrane suspension preparation

In accordance with the tenets laid out in the Declaration of Helsinki and after having properly acquired informed consent, human AM was obtained during elective cesarean deliveries and processed as reported in a prior study [7]. In brief, AM was obtained from the placenta, which exhibited negative serological test results for the human immunodeficiency virus, hepatitis B and C viruses, and syphilis. The placentas were maintained in sterile plastic bags on ice during transfer, and were handled via sterile techniques at all times. Under a lamellar-flow hood, the placentas were rinsed several times to remove excessive blood clots, with 0.9% normal saline containing 50 μg/ml of penicillin, 50 μg/ml of streptomycin, 100 μg/ml of neomycin, and 2.5 μg/ml of amphotericin B. The AM was separated from the remaining chorion via blunt dissection with two sets of forceps while immersed in the above-mentioned antibiotic-containing Earle’s balanced salt solution (Gibco BRL Life Technologies, Gaithersburg, MD). After being separated from the chorion, the AM was sliced into smaller pieces with Stevens scissors.

The methods for extraction of the proteins from the AM are as given below. First, employing sterile techniques, a homogenizer was used. After grinding the membrane, a sonicator was applied for additional protein extraction. Finally, the liquefied AM suspension was lyophilized to prevent damage to the AM suspension due to a prolonged preservation period and to maintain the bioactivity of the suspension.

The powdered AM was dissolved with Dulbecco's Modified Eagle Medium: F-12 medium (1:1; Jeil Biotechservices Inc., Daegu, ROK) and centrifuged for 10 min at 9,300× g. The supernatant was then separated carefully and diluted to 30% with PBS (Amgen, Thousand Oaks, CA).

### Rat serum preparation

Rat serum (Sigma, St. Louis, MO) was purchased and placed into a bottle coated with a substance that cuts out ultraviolet light. The bottles were diluted to 30% with PBS. The bottles were maintained in a refrigerator at 4 °C while in use.

### Alkali burn animal model

Thirty male Sprague-Dawley rats (weight range, approximately 250–300 g) were used in this study. All of the animals were treated in accordance with the guidelines laid out in the Association for Research in Vision and Ophthalmology (ARVO) Statement for the Use of Animals in Ophthalmic and Vision Research, and were approved by the Committee for Animal Research, Catholic University of Medicine. The rats were deeply anesthetized via the intraperitoneal injection of 50 mg/kg of tiletamine plus zolazepam (Zoletil; Virbac, Carros, France) and 15 mg/kg of xylazine hydrochloride (Rompun; Bayer, Leuverkeusen, Germany). Alkali injuries to the right eyes were induced via 60 s of exposure of the central cornea to a 4 mm diameter disk of filter paper soaked in 1 N NaOH, followed by rinsing with sterile saline (10 ml). The animals were then randomly allocated into three treatment groups. The control group (n=10) was treated topically with PBS, four times a day, immediately after alkali injury, for a total period of 2 days. The first study group (n=10) was treated topically with a 30% AM suspension, and the second study group (n=10) was treated topically with a 30% rat serum. Via slit lamp biomicroscopy (SL-15; kowa, Tokyo, Japan), the injured corneas were then evaluated and scored as described in detail below. Photographs of fluorescein-stained corneas at initial wounding and 48 h after the induction of the alkali burn were obtained to measure the area of the epithelial defect. The area of the corneal scrape wound was quantified from the photographs using a computer-assisted image analyzer (Image J 1.38x; National Institutes of Health, Bethesda, MD). The extent of healing was determined by the ratio of the difference between the zero hour and the remaining wound areas after 48 h.

### Scoring for corneal opacity and neovascularization

A previously described scoring system [8] was employed to measure the degree of corneal opacification between 0 to 5+: 0=clear and compact cornea; 1+=minimal superficial opacity; 2+=mild deep (stromal) opacity with pupil margin and iris vessels visible; 3+=moderate stromal opacity with only pupil margin visible; 4+=intense stromal opacity with anterior chamber visible; 5+=maximal corneal opacity with total obscuration of the anterior chamber. Corneal neovascularization (NV) was graded between 0 and 3 per corneal quadrant, with increments of 0.5, using a grid system based on the centripetal extent of neovascular branch outgrowth from the corneoscleral limbus [9]. Dilated limbal vessels not penetrating the corneal stroma were not considered representative of corneal NV. All gradings were conducted in a masked fashion. The scores for each quadrant were then summed to derive the corneal NV indices (range, 0–12) for each eye at a given time point [9]. The mean differences in corneal opacities and corneal NV scores were compared among each group.

### Histologic documentation of inflammatory cell infiltration

The 30 rats were killed after the clinical examination, and for histological evaluation, three eyes in each group were randomly selected and enucleated and fixated in 4% formaldehyde. Corneas were sectioned centrally and embedded in paraffin, and 5 μm sections were stained with hematoxylin and eosin. Next, for immunohistochemical evaluation, the tissues were fixed for 20 min at room temperature (RT) and blocked for 1 h with 2% bovine serum albumin. Rat anti-F4/80 antibody (a macrophage marker) was employed to evaluate the acute inflammatory reaction; this antibody (1:1,000; Sigma Aldrich, St.Louis, MO) was administered and incubated for 1 h at RT. The slides were washed three times with PBS and incubated for 1 h at RT with goat-anti-mouse-Alexa 546 (Invitrogen, Carlsbad, CA). The cells were rinsed with PBS, and the nuclei were counterstained with Hoechst 33342 (1:500) for 10 min at RT. The samples were observed under a fluorescence microscope (Axiovert 200; Carl Zeiss Inc., Göttingen Germany). For the comparison of these study samples with the natural state of the cornea, the rat corneas without alkali burns were treated via the same method as described above.

### Statistical analysis

We confirmed all observations via several different experiments. The data were compared with each group and expressed as means±standard deviation (SD). All statistical data were analyzed via one-way Analysis of Variance (SPSS 13.0) and Bonferonni’s multiple comparisons; results were considered significant at p<0.05.

## Results

### Corneal epithelial wound healing in alkali burn

**Figure 1:**
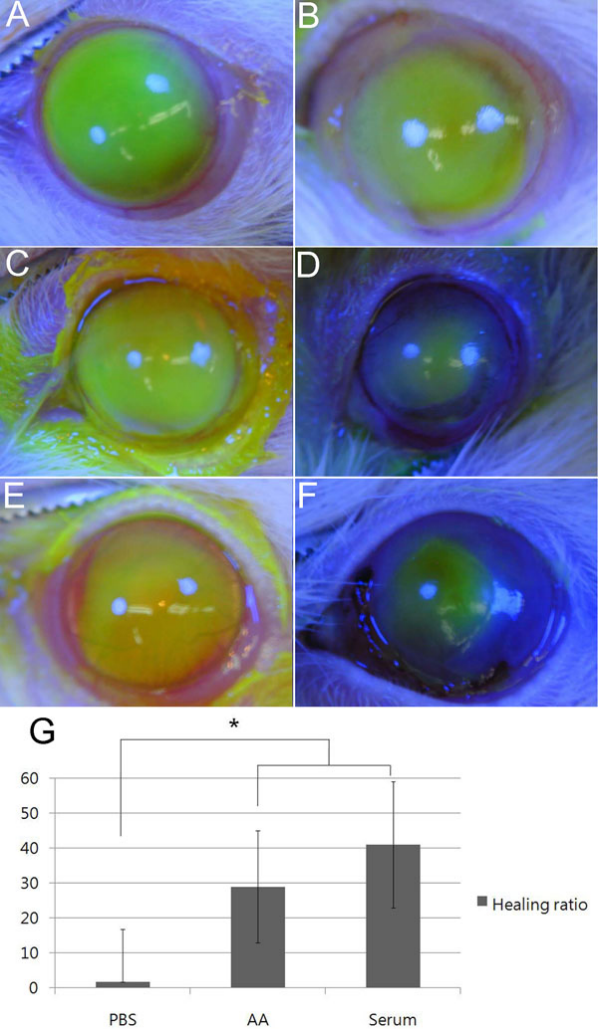
Corneal epithelial wound healing immediately and 48 h after alkali burn. **A**: The control group, immediately after alkali burn. **B**: The control group, 48 h after alkali burn. **C**: AM suspension group, immediately after alkali burn. **D**: AM suspension group, 48 h after alkali burn. **E**: Serum eyedrop group, immediately after alkali burn. **F**: Serum eyedrop group, 48 h after alkali burn. The figure is representative of the experiments. In the control group, the wound immediately after alkali burn (**A**) was not changed after 48 h (**B**). However, in the AM suspension group, the wound immediately after alkali burn (**C**) was decreased after 48 h (**D**). Furthermore, in the serum eyedrop group, the wound immediately after alkali burn (**E**) was decreased after 48 h (**F**). The wound healing ratio (**G**) was significantly higher in the AM suspension and serum eyedrop groups than the control group (p<0.001). The difference between the AM suspension and serum eyedrop groups was not statistically significant (p=0.458).

Figure 1 shows corneal epithelial wound healing immediately and 2 days after alkali burn in the control (PBS) group, AM suspension group, and serum eye drop group; the results shown are representative of the experiments. In the control group (Figure 1A,B), 1.8±5.1% of the alkali burn wound was recovered, whereas the AM suspension (Figure 1C,D) and serum eyedrop groups (Figure 1E,F) evidenced wound healing rates of 34.1±7.7% and 41.5±6.1%, respectively. The control group exhibited a wound healing rate that was significantly lower than that of the other two study groups (p<0.001), and the difference between the AM suspension and serum eyedrop group was not found to be statistically significant. (p=0.458; Figure 1G).

### Corneal opacity and neovascularization

**Figure 2:**
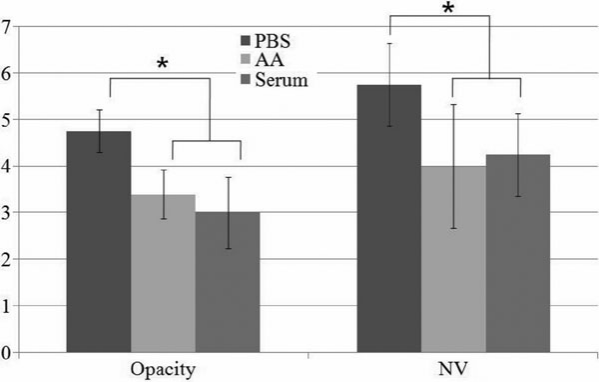
Corneal opacity and neovascularization (NV) scores 48 h after alkali burn. In the opacity score, the amniotic membrane (AM) suspension and serum eyedrop groups showed significantly lower scores compared to the control group (p<0.001). In addition, in the NV score, the AM suspension and serum eyedrop groups also showed significantly lower scores compared to the control group (p=0.006).

The scores for corneal opacity developing after alkali burn were 4.75±.46, 3.40±.51, and 3.00±.76 in the control, AM suspension, and serum eyedrop groups, respectively (Figure 2). The control group exhibited a corneal opacity score that was significantly higher than those of the other two study groups (p<0.001), while the difference between the AM suspension and serum eyedrop groups was not found to be statistically significant (p=0.491). Additionally, the scores for the NV developed after alkali burn were 5.75±.89, 4.00±.33, and 4.25±.88 in the control, AM suspension, and serum eyedrop groups, respectively. The control group evidenced a corneal opacity score that was significantly higher than those of the two study groups (p=0.006), while the difference between the AM suspension and serum eyedrop groups was not found to be statistically significant (p>0.05).

### Histologic documentation of inflammatory cell infiltration

**Figure 3:**
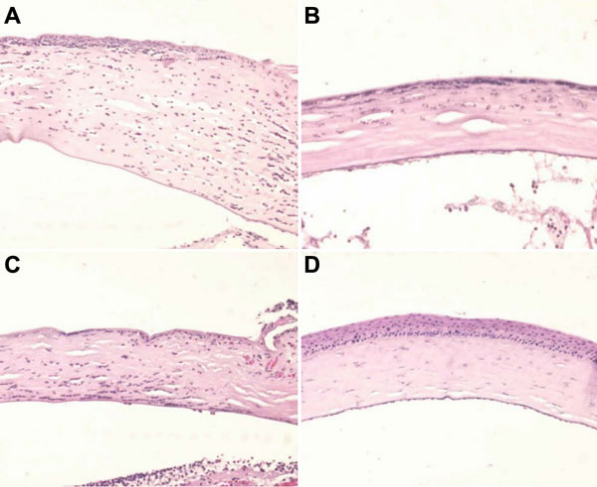
Light microscopic findings 48 h after alkali burn at 100× magnification. **A**: The control group and **B**: amniotic membrane (AM) suspension group. **C**: Serum eyedrop group and **D**: comparison group without any manipulation. The figure is representative of the experiments. The corneal thickness was greatly increased and many polymorphonuclear leukocytes infiltrated into the corneal stroma in the control group (**A**). However, relatively fewer polymorphonuclear leukocytes infiltrated into the cornea in the AM suspension (**B**) and serum eyedrop groups (**C**). Note that the comparison group without any manipulation (**D**) shows normal corneal thickness without any polymorphonuclear leukocytes infiltration.

**Figure 4:**
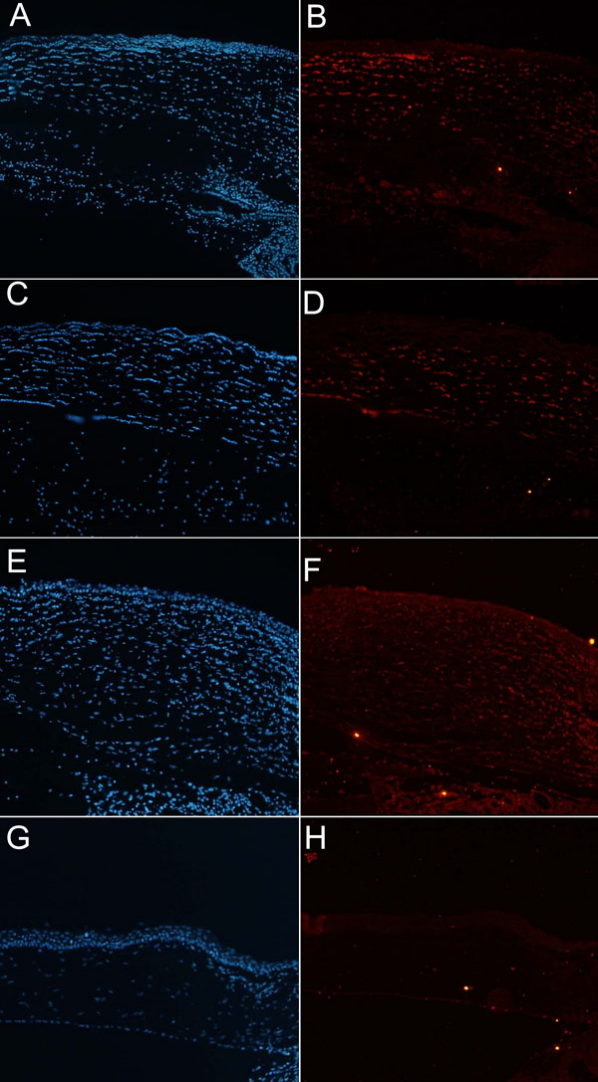
Immunohistochemical staining 48 h after alkali burn at 100× magnification. **A**, **B**: The control group; **C**, **D**: the amniotic membrane (AM) suspension group; **E**, **F**: the serum eyedrop group; and **G**, **H**: the comparison group without any manipulation. The figure is representative of the experiments. Cell nuclei were stained with 4',6-diamidino-2-phenylindole (DAPI; blue; **A**, **C**, **E**, **F**), and the F4/80 expressions (red) on the section of cornea 48 h after alkali burn were noted (**B**, **D**, **F**, **H**) The amount of F4/80 expression was largest in the control group. However, F4/80 expression is hardly seen in the AM suspension and serum eyedrop groups. Note that there is no expression of F4/80 in the comparison group without any manipulation.

**Figure 5:**
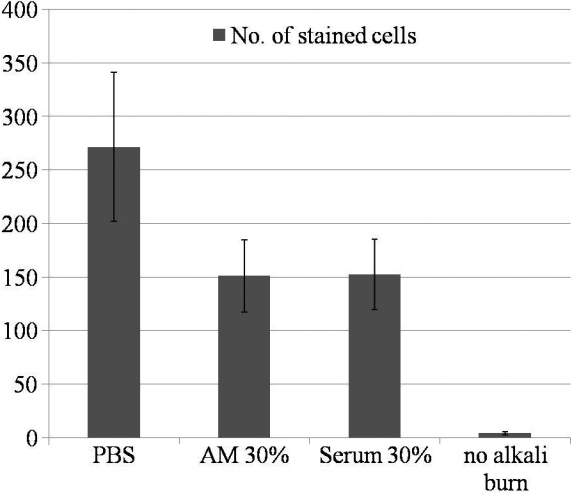
Numbers of stained cells expressing F4/80 on the section of cornea 48 h after alkali burn. The number of stained cells was significantly higher in the control group compared to the AM suspension and serum eyedrop group. (p=0.027) Note there were almost no stained cells in the comparison group without any manipulation.

Light microscopic findings in the control, AM suspension, serum eyedrop, and comparison groups without any manipulation 48 h after alkali burn are shown in Figure 3. The corneal thickness was greatly increased and many polymorphonuclear leukocytes infiltrated into the corneal stroma in the control group (Figure 3A). However, relatively fewer polymorphonuclear leukocytes infiltrated into the cornea in the AM suspension (Figure 3B) and serum eyedrop groups (Figure 3C) compared to the control group. In addition, the corneal thicknesses were slightly increased in the AM suspension (Figure 3B) and serum eyedrop groups (Figure 3C) compared to the comparison group without any manipulation (Figure 3D). In immunocytochemistry for mouse F4/80 antigen, large quantities of F4/80+ cells were noted in the control group (Figure 4A,B), particularly in the corneal limbal region. By way of contrast, we noted minimal F4/80 expression in the AM suspension (Figure 4C,D) and serum eyedrop groups (Figure 4E,F). Numbered stained cells were 163.7±1.2, 76.4±1.8, 87.0±9.0, 7.3±.8 in the control, AM suspension, serum eyedrop, and comparison group without any manipulation (p=0.027; Figure 5).

## Discussion

Failure of the ocular surface occurs in a broad range of clinical conditions, with differing pathogeneses. Ocular surface diseases also frequently prove to be nonresponsive to conventional treatments, including topical therapy (ocular lubricants), therapeutic contact lenses, phototherapeutic lasers, medical (botulinum toxin) or surgical (stitching the lids together) tarsorrhaphy, protective glasses, etc [10]. In the treatment of nonresponsive ocular surface disease, serum eyedrops, AM transplantation, and limbal epithelial stem cell grafts represent three recent advances [1]. Among these emerging tools, serum eyedrops harbor an abundance of epitheliotropic factors present in tears, and these eyedrops are widely employed in the treatment of dry eye disease [10]. Despite their proven efficacy [10,11] in dry eye disease, however, topical autologous serum eyedrops may be limited in several ways. Fox et al. [12] previously reported and described a case of scleral vasculitis in a patient with rheumatoid arthritis that arose after serum eyedrop treatment. Conjunctivitis was also found in other case reports [13,14]. Conjunctivitis may prolong or aggravate ocular infections because serum contains an abundance of nutrients, as shown in cases of *Candida* infectious crystalline keratopathy and coagulase-negative staphylococcus keratitis in patients with persistent epithelial defects following serum eyedrop treatment [15]. Additionally, owing to its immunogenicity, only autologous serum can be employed; this precludes its use in patients with hemoglobinopathies, elevated total protein and albumin levels, or taking medications that may be injurious to the cornea, as well as in patients with positive microbial serology [10].

The use of topical steroids profoundly reduces inflammatory reactions, but interferes with the process of wound repair, thereby exacerbating the risk of corneal ulceration and perforation [16]. However, AM has both epithelialization and anti-inflammation properties; this has been demonstrated in previous clinical studies concerning AM transplantation [5-7]. Furthermore, AM has been shown to possess antibacterial properties [17,18]. Gicquel et al. [19] reported previously that early AM transplantation can be employed as a safe adjuvant therapy during antibacterial treatment in cases of severe bacterial keratitis. Therefore, if AM is prepared in eyedrop form and maintains its effects, it may constitute a potent clinical tool for the treatment of ocular surface disease. In our previous study, the authors demonstrated that the AM suspension exerts a positive effect on corneal re-epithelialization according to its concentration in a simple in vitro wound healing model [20]. In the present study, we attempted to show that the anti-inflammatory effect of AM is maintained when AM is applied in suspension form in an animal model.

To prepare the inflammatory wound model, we induced an alkali injury to the rat corneas using 1 N NaOH. The concentration (30%) of the AM suspension was determined from the results of our previous study [20], and the concentration of rat serum was set to 30%, which was identical to that of the AM suspension, to compare the actual effects of the two eyedrops.

In terms of re-epithelialization, both the AM suspension and serum eyedrop groups evidenced significantly more rapid healing than was observed in the control group (Figure 1). Additionally, with regard to its anti-inflammatory effect, the AM suspension and serum eyedrops suppressed acute inflammatory reactions, as was revealed by the results of immunochemical assays using F4/80 (mouse macrophage marker; Figure 4). Lower F4/80 expression was noted in the AM suspension and serum eyedrop group relative to the control group, which evidenced relatively high F4/80 expression. These groups also evidenced significantly suppressed corneal opacity and NV relative to the control group (Figure 2). In our pilot study before the full-scale experiment, the epithelialization was almost completed in both study groups at approximately 48 h after the alkali burn. Therefore, we established a follow-up period of 48 h to evaluate both epithelialization and anti-inflammation effects. However, to assess late sequelae of the chemical burns, studies with longer follow-up periods are warranted.

Although the AM suspension exhibited a significantly rapid healing rate relative to the control group, we noted no significant differences between the serum eyedrop and AM suspension groups. In this study, commercial sterile rat serum was used to prevent immunogenicity, whereas AM was obtained from humans. The xenogenicity of the AM suspension might have had some influence on the results. Additionally, substances that modulate epithelialization are abundant in serum eyedrops, as well as in AM suspension [10]. In this study, serum treatment was also shown to ameliorate acute inflammatory reactions as in AM suspension. Therefore, in addition to its epitheliotropic effect, serum appears to exert some anti-inflammatory effects, although the mechanism underlying the anti-inflammatory effects of serum have yet to be elucidated.

In conclusion, the AM suspension used herein was determined to enhance epithelial healing and reduce corneal opacity and NV in inflammatory corneal wounds; it also suppressed the expression of F4/80 in the corneal stroma, thereby indicating that the suspension form of AM maintains anti-inflammatory effects comparable to those of serum eyedrops. With its non-immunogenicity and relatively low risk of possible infection, AM suspension appears to constitute a valuable clinical technique for the treatment of symptoms associated with intractable inflammatory ocular surface disease, including severe dry eye, chemical burns, and persistent epithelial defects.
